# Expression and contribution to virulence of each polysaccharide capsule of *Bacillus cereus* strain G9241

**DOI:** 10.1371/journal.pone.0202701

**Published:** 2018-08-22

**Authors:** Jennifer M. Scarff, Yuliya I. Seldina, James M. Vergis, Christy L. Ventura, Alison D. O’Brien

**Affiliations:** Department of Microbiology and Immunology, Uniformed Services University of the Health Sciences, Bethesda, MD, United States of America; University of Texas Medical School at Houston, UNITED STATES

## Abstract

*Bacillus cereus* strain G9241 was isolated from a patient with pneumonia who had an anthrax-like illness. Like *Bacillus anthracis*, the virulence of G9241 is dependent on two large plasmids. In G9241 those plasmids are pBCXO1 and pBC210. There is a multi-gene capsule locus on each of these virulence plasmids, and both capsules are produced by G9241 *in vitro* and in mice. The *hasACB* operon on pBCXO1 is responsible for production of a hyaluronic acid (HA) capsule. The locus on pBC210 encodes a putative tetrasaccharide (TS) capsule that assembles in a Wzy-dependent manner. We found that the pBC210 capsule locus is transcribed as two operons and identified the promoter regions responsible for transcription. We constructed isogenic mutants to assess the role of genes in the two TS capsule operons in production of the capsule. Spores of strains deficient in production of either the HA or TS capsule were inoculated subcutaneously or intranasally into A/J and C57BL/6 mice to determine the lethal dose 50% of each bacterial mutant by each route of infection. The loss of the HA capsule attenuated G9241 more than the loss of the TS capsule for both infection routes in both mouse strains. Overall, our data further characterize the unique TS capsule on pBC210 and demonstrate that the two capsules do not have the same impact on virulence of G9241.

## Introduction

The *Bacillus cereus sensu lato* group consists of both *B*. *cereus* and *Bacillus anthracis*. *B*. *cereus* is typically associated with food poisoning or infections in immunocompromised patients while *B*. *anthracis* is the etiologic agent of anthrax [1]. There are two virulence plasmids in *B*. *anthracis* which are critical to disease progression, pXO1 and pXO2. The toxigenic plasmid, pXO1, encodes protective antigen (PA), lethal factor (LF), edema factor (EF), and virulence regulator AtxA [2–5]. The operon required for production of the poly-γ-D-glutamic acid (PDGA) capsule is encoded on pXO2 [6].

*B*. *cereus* strain G9241 was isolated from a patient who suffered from an anthrax-like respiratory infection. G9241 contains two virulence plasmids. One of these, pBCXO1, is highly similar to pXO1 and also encodes PA, LF, EF, and AtxA1 while the other plasmid, pBC210 (formerly pBC218), is unique compared to pXO2 [7]. In fact, pBC210 encodes a PA homolog (PA2), a novel ADP-ribosylating toxin, certhrax, and an AtxA homolog, AtxA2 [7–10]. Each virulence plasmid also contains genetic loci that encode the proteins to construct a polysaccharide capsule. Plasmid pBCXO1 carries the *hasACB* operon that is responsible for production of a hyaluronic acid (HA) capsule, while pBC210 contains a capsule locus that is required for elaboration of a putative tetrasaccharide (TS) capsule [7].

The two capsules in G9241 are both produced *in vivo* [11, 12]. The HA capsule is regulated by AtxA1, and the TS capsule is regulated by either AtxA1 or AtxA2 [12]. The *hasACB* operon on pBCXO1 is similar to the *hasABC* operons in *Streptococcus pyogenes* strains and is responsible for the production of an HA capsule, a disaccharide of glucuronic acid and N-acteylglucosamine. The HasA synthase assembles the high molecular weight repeating disaccharide polymers, and HasC and HasB are involved in synthesis of the nucleotide sugar precursors [13].

The capsule locus on pBC210 was initially characterized as a fifteen gene locus responsible for production of the TS capsule [7]. The final nine genes in the capsule locus were later identified as *bpsX*-*H* [11]. The TS capsule locus is similar in content to the Wzy-dependent polysaccharide capsules of *Streptococcus pneumoniae* [14]. The Wzy-dependent *S*. *pneumoniae* capsule loci encode four regulatory proteins, a glycosyltransferase for each sugar in the subunit, a flippase, a polymerase, and enzymes required to synthesize sugars that are unique to the capsule. The regulatory proteins are conserved among all serotypes, and the other proteins are serotype-specific [14, 15]. The synthesis of these types of capsules is initiated by the successive addition of each sugar subunit to a carrier molecule on the interior surface of the bacterial membrane. The Wzx flippase then “flips” the sugar moiety to the outer surface of the membrane and Wzy polymerase transfers the already formed chains on the surface to the recently “flipped” subunit.

Other *B*. *cereus* strains have emerged in the Southeastern United States that caused a severe anthrax-like respiratory illness in metalworkers or a cutaneous infection in a healthy adult man [16–20]. These strains are encapsulated but do not contain pXO2-like plasmids or the PDGA capsule locus [18, 21, 22]. However, the strains do contain pBCXO1-like plasmids that encode an HA capsule in addition to the PA, LF, and EF associated with *B*. *anthracis* [19, 21, 23]. Only about half of the isolated strains have been either PCR- or sequence-confirmed to have pBC210 or the TS capsule locus [19, 21, 22]. Although one strain, BcFL2013, isolated from the cutaneous infection, has a partial plasmid of pBC210 that does not include the TS capsule locus [23].

In addition to these emerging U.S. strains, isolates of *B*. *cereus* biovar *anthracis* have been found as the sources of fatal infections in great apes in Cameroon and Côte d’Ivoire and wild and domestic animals in the Central African Republic and the Democratic Republic of the Congo [24, 25]. Only the strains from the great apes in Cameroon and Côte d’Ivoire have been further genetically characterized. These strains harbor both pBCXO1 and a pXO2-like plasmid, pBCXO2. Consequently, these strains produce PA, LF, and EF as well as the HA and PDGA capsules [26, 27].

In this study, we used G9241 as a prototypical U.S. emergent strain of *B*. *cereus* with *B*. *anthracis*-associated virulence factors. We determined that the unique pBC210 TS capsule locus is comprised of two operons and identified transcriptional start sites and promoter regions for each operon. We also demonstrated that deletion of a glycosyltransferase or the polymerase from the TS capsule locus abrogated production of the capsule. Lastly, we compared the virulence of G9241 and two isogenic mutants that produced only HA or TS capsules and determined that the HA capsule has a larger role in virulence of G9241 when compared to the TS capsule.

## Results

### Genetic characterization of pBC210 TS capsule locus

**Fig 1:**
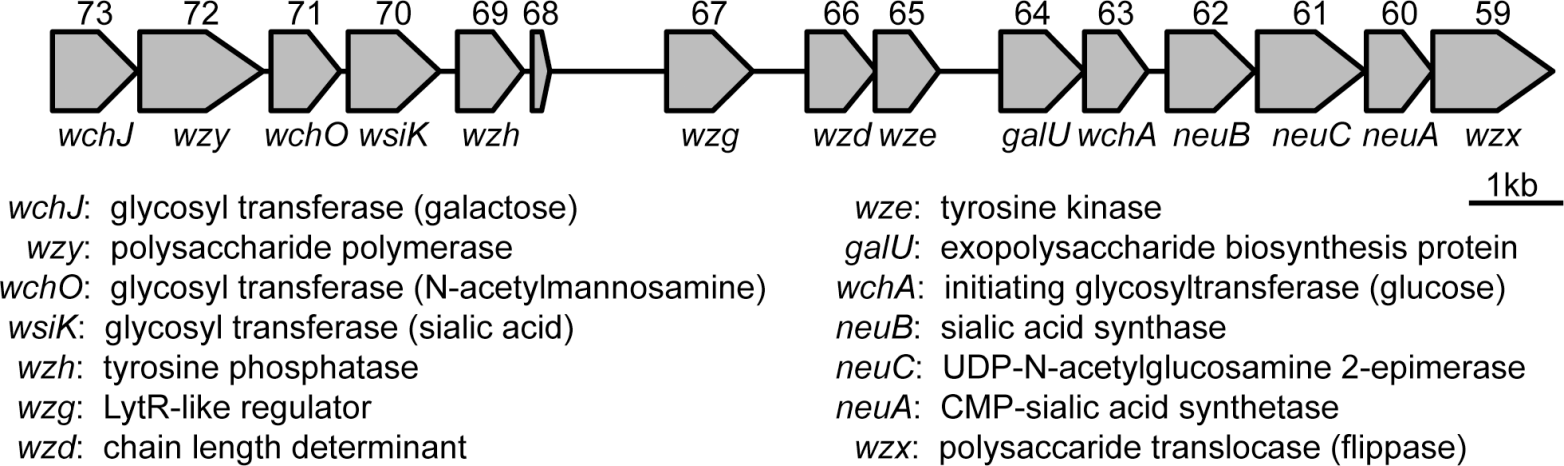
A linear representation of the pBC210 capsule locus and the function of the encoded proteins. The original locus numbers for the genes (BCE_G9241_pBC218_00XX) are above the genes. The nomenclature that is consistent with Wzy-dependent capsules from *S*. *pneumoniae* is below each gene. The protein function for each gene product, as initially annotated or determined by homology, is listed.

The TS capsule locus was initially identified as a fifteen gene region on pBC210 (BCE_G9241_pBC218_0073 to BCE_G9241_pBC218_0059) [7]. The locus contains genes associated with Wzy-dependent capsule loci that have some similarity to LPS assembly loci in Gram-negative bacteria. Consequently, we switched the original nomenclature for the genes in the TS capsule locus on pBC210 [7] to one that is universally used for proteins of similar functions between capsule and LPS loci. That nomenclature is the accepted paradigm to describe genes in the capsule loci of another Gram-positive bacterium, *S*. *pneumoniae* [15]. One gene in the original locus (G9241_pBC218_0068) encoded a short protein with no known homologs or conserved domains, so we omitted that gene from our final capsule locus genetic description. The nomenclature and predicted functions for the other fourteen genes in the TS capsule locus are presented in Fig 1. The capsule is defined as a Wzy-dependent capsule locus based on the sequence homology of genes with those that encode Wzy polymerase and Wzx flippase. In addition to these two genes, the *wzh*, *wzg*, *wzd*, and *wze* genes that are conserved among *S*. *pneumoniae* capsule loci are also present in the G9241 capsule locus. The pBC210 capsule locus encodes four putative glycosyltransferases. The initiating glycosyltransferase in G9241 is homologous to *wchA* in *S*. *pneumoniae*, a gene whose product adds a glucose to the undecaprenyl phosphate carrier molecule. The other three glycosyltransferases are predicted to add each successive sugar to the repeat unit in an undetermined order. While we do not know the structure of the TS capsule, we can deduce the likely nature of the sugar subunits based on homology to glycosyltransferases of known function. The two glycosyltransferases with homologs in *S*. *pneumoniae* are WchJ, a galactosyltransferase, and WchO, an N-acetylmannosaminyltransferase. The fourth transferase is homologous to CpsK, a sialic acid transferase from *Streptococcus agalactiae*. As Cps is not a specific descriptor for the protein function and with no known *S*. *pneumoniae* homolog, we have designated this transferase WsiK. The TS locus also has four genes that encode proteins predicted to synthesize sugars for the capsule. These genes are *galU* and the sialic acid synthesis genes *neuA*, *neuB*, and *neuC*.

### Transcriptional characterization of capsule locus on pBC210

**Fig 2:**
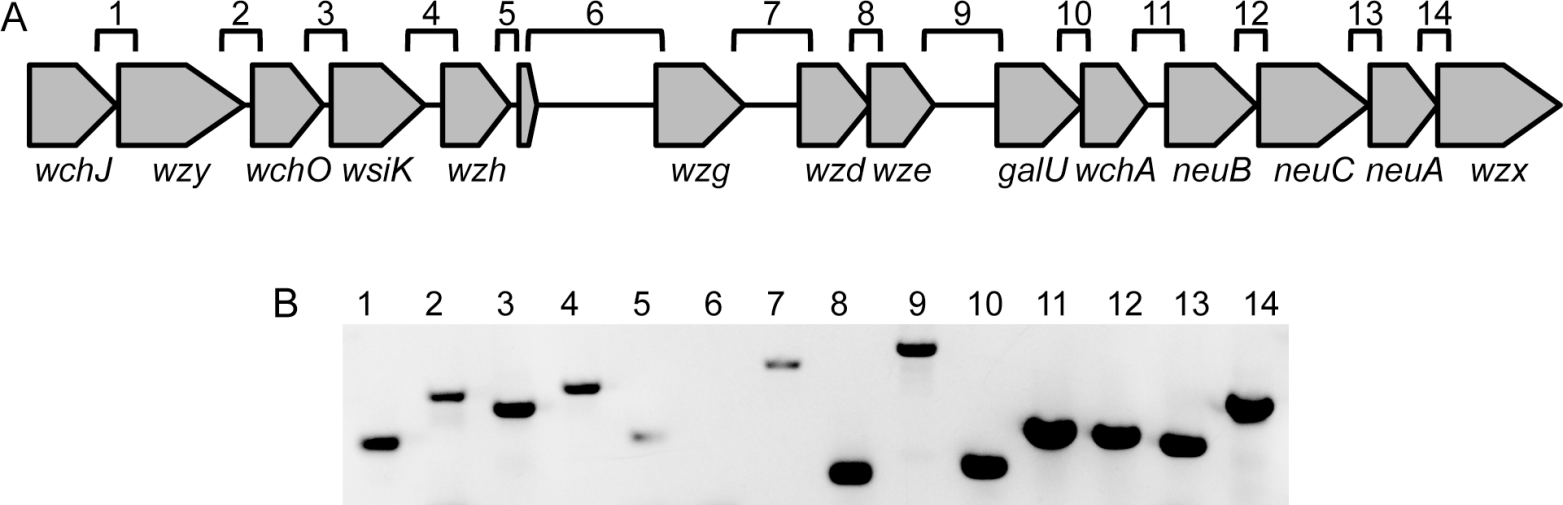
TS capsule locus has two potential operons. RNA was isolated from G9241 and used to synthesize cDNA. To determine whether transcripts included multiple genes, the cDNA was used to assay for the junctions between the genes (A) by PCR (B).

Although the pBC210 capsule locus has similar genes to the capsule operons of many *S*. *pneumoniae* strains, the genes are not organized in a similar manner. Additionally, the *S*. *pneumoniae* capsule loci have small to no gaps between the genes and are transcribed as a single operon [28, 29]. Contrarily, the capsule locus in pBC210 contains three gaps between genes that are over 500 bp, and one of those gaps is over 1 kb. To determine whether this unique capsule locus is an operon, we used cDNA as a template for PCR amplification of the junctions between genes in the capsule locus (Fig 2A). The presence of a PCR result product at a gene junction indicates that the genes are transcribed together, whereas the absence of a PCR product at a gene junction implies that those two genes are not transcribed together. We were able to detect the gene junctions for the first five genes in the locus, *wchJ* to *wzh*, as well as the junctions for the last nine genes in the locus, *wzg* to *wzx* (Fig 2B). We were unable to detect the junction between *wzh* and *wzg*. These data indicated that there are two potential operons in the TS capsule locus.

**Fig 3:**
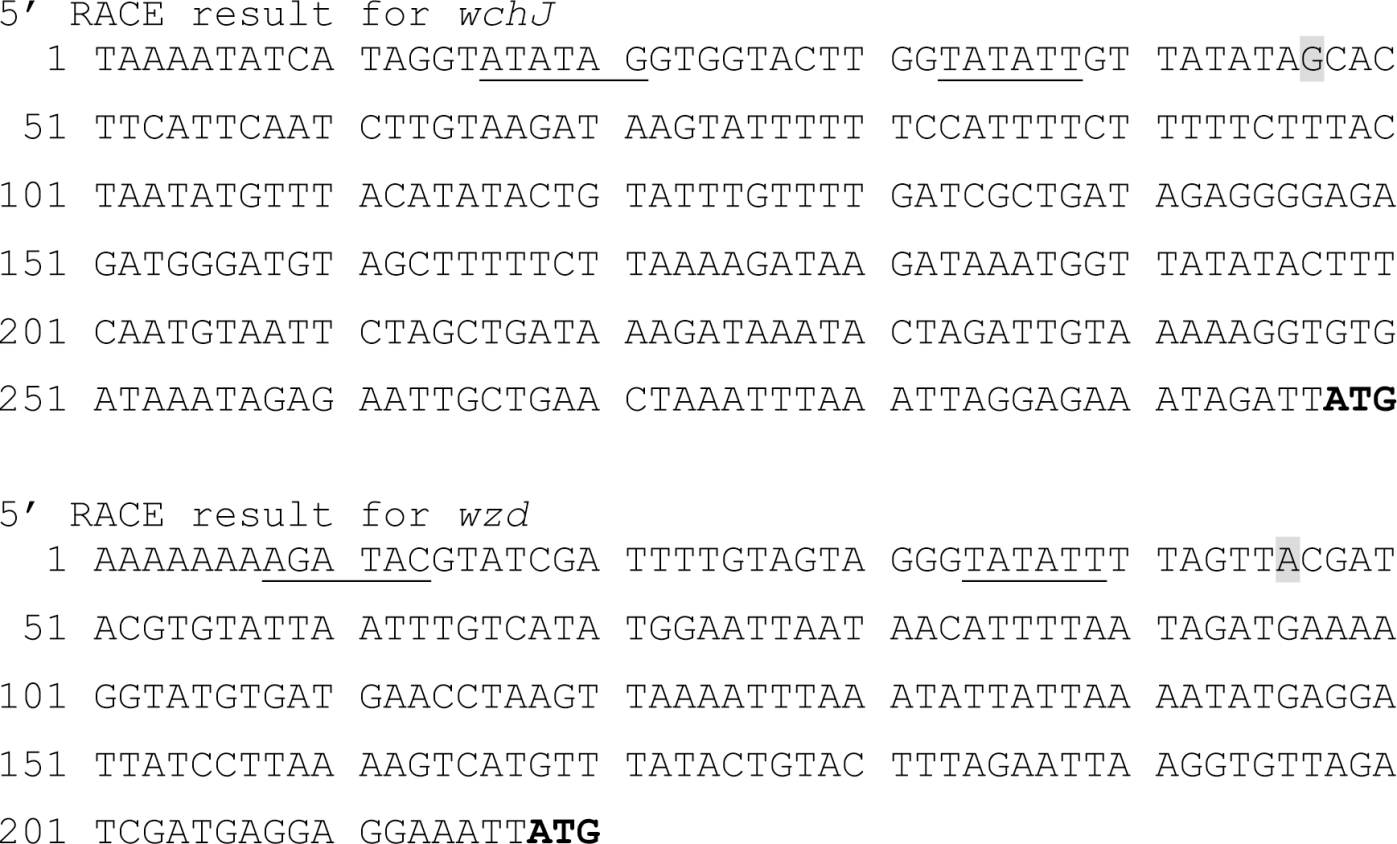
5’ RACE results for *wchJ* and *wzd*. The transcriptional start sites, highlighted in gray, determined by 5’RACE are 251 bp for *wchJ* (A) and 172 bp for *wzd* from the ATG start codon, in bold. Potential -10 and -35 boxes from these start sites are underlined.

To further investigate the two potential transcripts for the TS capsule locus, we used 5’ RACE to map transcriptional start sites. The transcriptional start site for *wchJ* was determined to be 251 bp from the start codon (Fig 3). We were unable to determine a transcriptional start site for *wzg*. Consequently, we also investigated the next gene in the second potential transcript for a transcriptional start site and determined that the *wzd* transcript started 172 bp from the start codon (Fig 3).

**Fig 4:**
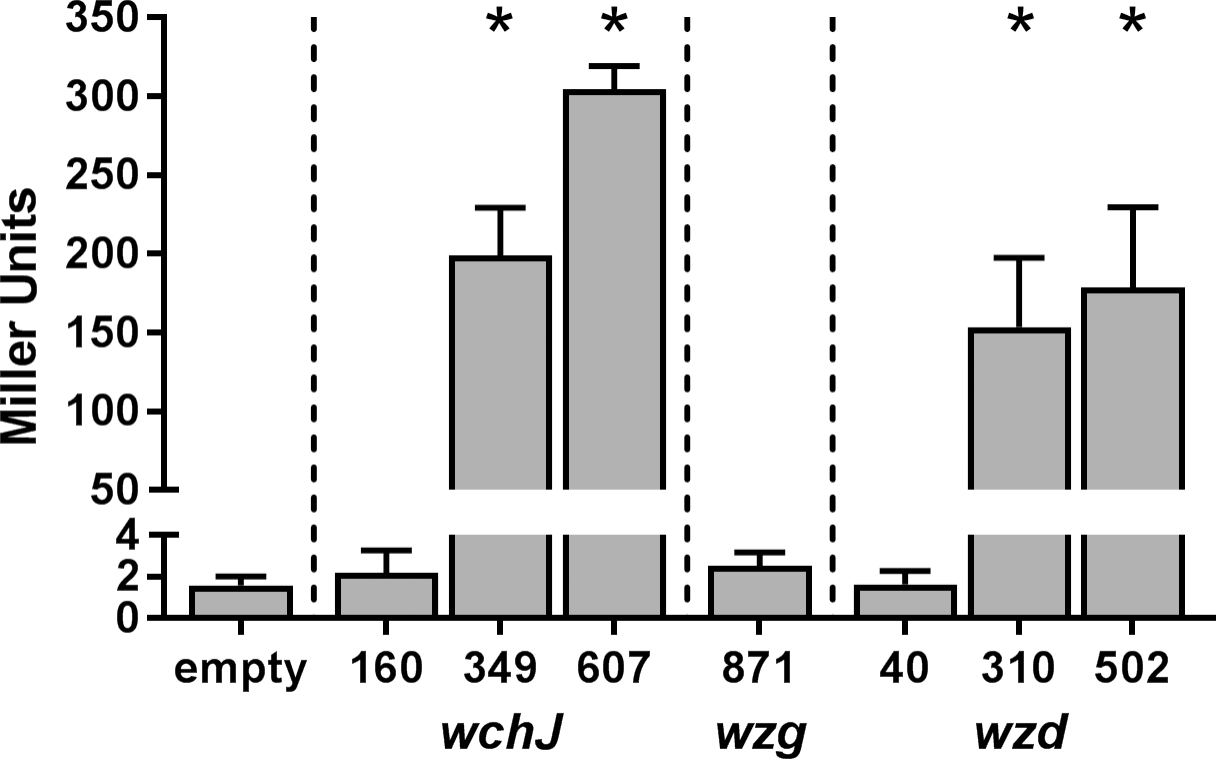
Promoter activity for *wchJ*, *wzg*, and *wzd*. Fragments proximal to the start codon for *wchJ* (160, 349, and 607 bp), *wzg* (871 bp), and *wzd* (40, 310, and 502 bp) were cloned before the promoterless *lacZ* in pHT304-18Z in strain Δ*wchA*Δ*hasACB*. The cells were grown under capsule-inducing conditions for 4 h, and the β-galactosidase activity was measured. The bars indicate the mean of four experiments and the error bars the standard deviation. *Significantly different from empty vector control as determined by one-way ANOVA with Dunnett’s multiple comparisons test (*P* < 0.0001).

We used promoter fusion assays to further investigate the transcriptional start sites. We cloned fragments that were upstream of the start codon for *wchJ*, *wzd*, and *wzg* into a vector with a promoterless *lacZ* and assayed for β-galactosidase activity after growth in capsule-inducing conditions (Fig 4). A 160 bp fragment that was smaller than the *wchJ* 5’ RACE product was unable to induce β-galactosidase activity in the assay. The 349 and 607 bp fragments for *wchJ* induced a significant increase of β-galactosidase activity compared to the empty vector control (*P* < 0.0001). Similarly, a 40 bp fragment that was smaller than the 5’ RACE product for *wzd* was unable to promote β-galactosidase activity. However, fragments of 310 and 502 bp for *wzd* induced a significant increase in β-galactosidase activity compared to the empty vector control (*P* < 0.0001). We were unable to detect any promoter activity for even the largest fragment of *wzg*, 871 bp to translational start site. We concluded that the TS capsule has two potential operons and at least two promoter sites that facilitate gene expression.

### Effect of capsule gene deletion on expression

**Fig 5:**
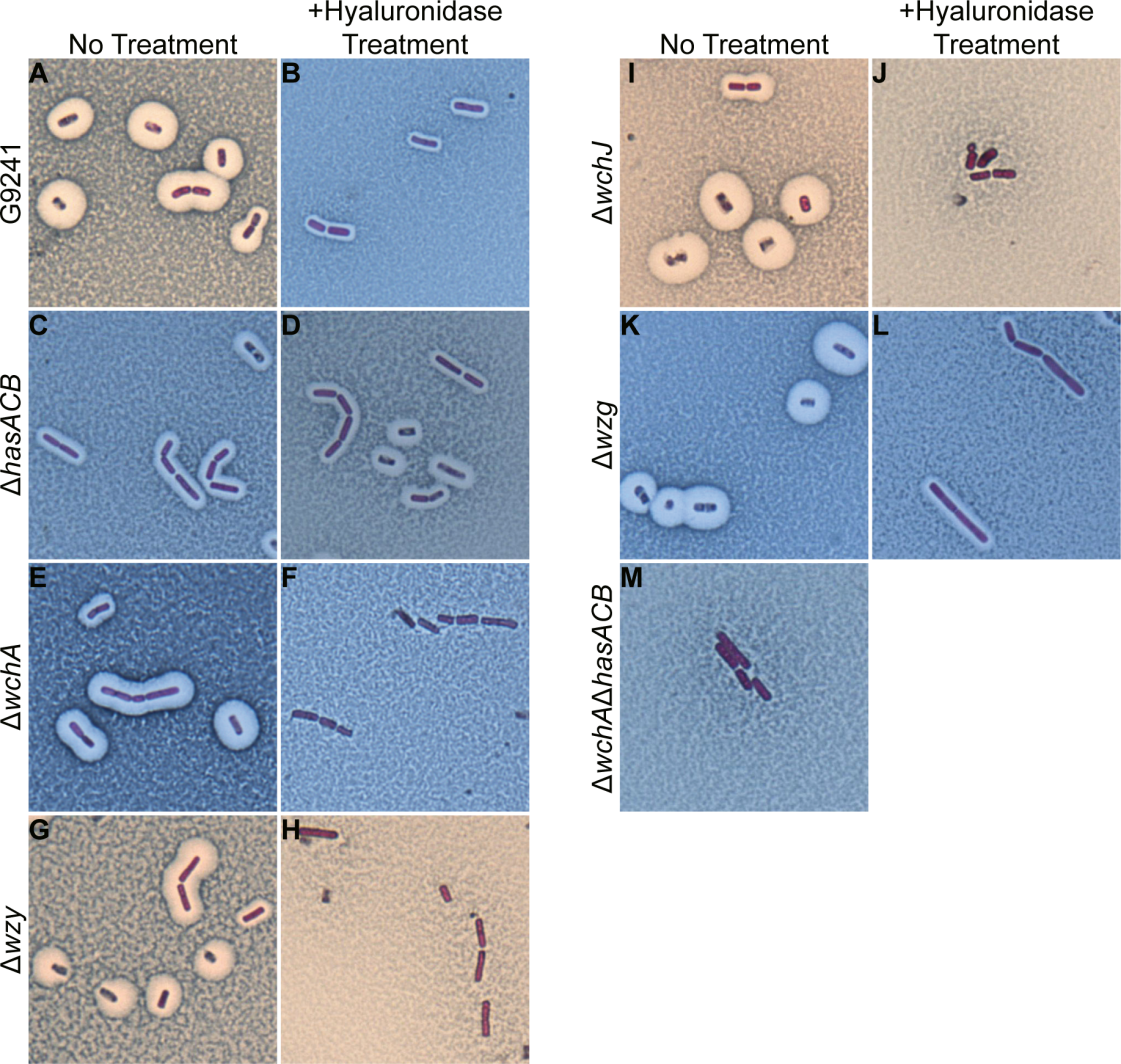
Capsule production by capsule mutants. Maneval stain of cells from 24 h capsule-inducing cultures. G9241 (A, B), Δ*hasACB* (C, D), Δ*wchA* (E, F), Δ*wzy* (G, H), Δ*wchJ* (I, J), Δ*wzg* (K, L), and Δ*wchA*Δ*hasACB* (M) cells were either untreated (A, C, E, G, I, K, M) or treated with 200 U of hyaluronidase (B, D, F, H, J, L) prior to application of Maneval stain. Cells were visualized with oil immersion at 1,000x magnification.

We made isogenic mutants of the *hasACB* operon and *wchA*, *wzy*, *wchJ*, *wzg* in the TS capsule locus and determined the capsule phenotype for these strains. As previously reported [11, 12], G9241 produces both the HA and TS capsules. The large halo around the cells with no treatment is indicative of the HA capsule (Fig 5A), and the smaller halo around the bacteria that remains after hyaluronidase treatment is the TS capsule (Fig 5B). The Δ*hasACB* mutant produced only the TS capsule, as indicated by the same sized halo around cells after no treatment (Fig 5C) and with hyaluronidase treatment (Fig 5D). Deletion of the initiating glycosyltransferase of the TS capsule, *wchA*, abrogated production of the TS capsule; the Δ*wchA* cells had a large halo representative of the HA capsule with no treatment (Fig 5E) and no halo remained after hyaluronidase treatment (Fig 5F). Similarly, deletion of the polymerase *wzy* (Fig 5G and 5H) or the glycosyltransferase *wchJ* (Fig 5I and 5J) also resulted in cells that produced only HA capsule, as evidenced by the loss of the halos around cells after hyaluronidase treatment. Deletion of the putative LytR-like regulator, *wzg*, had no effect on expression of either capsule; the Δ*wzg* cells had the large HA halo with no treatment (Fig 5K) and the smaller TS capsule remained visible after hyaluronidase treatment (Fig 5L). In the Δ*wchA*Δ*hasACB* mutant, neither capsule was produced, as indicated by the absence of a halo around cells that were untreated (Fig 5M).

**Fig 6:**
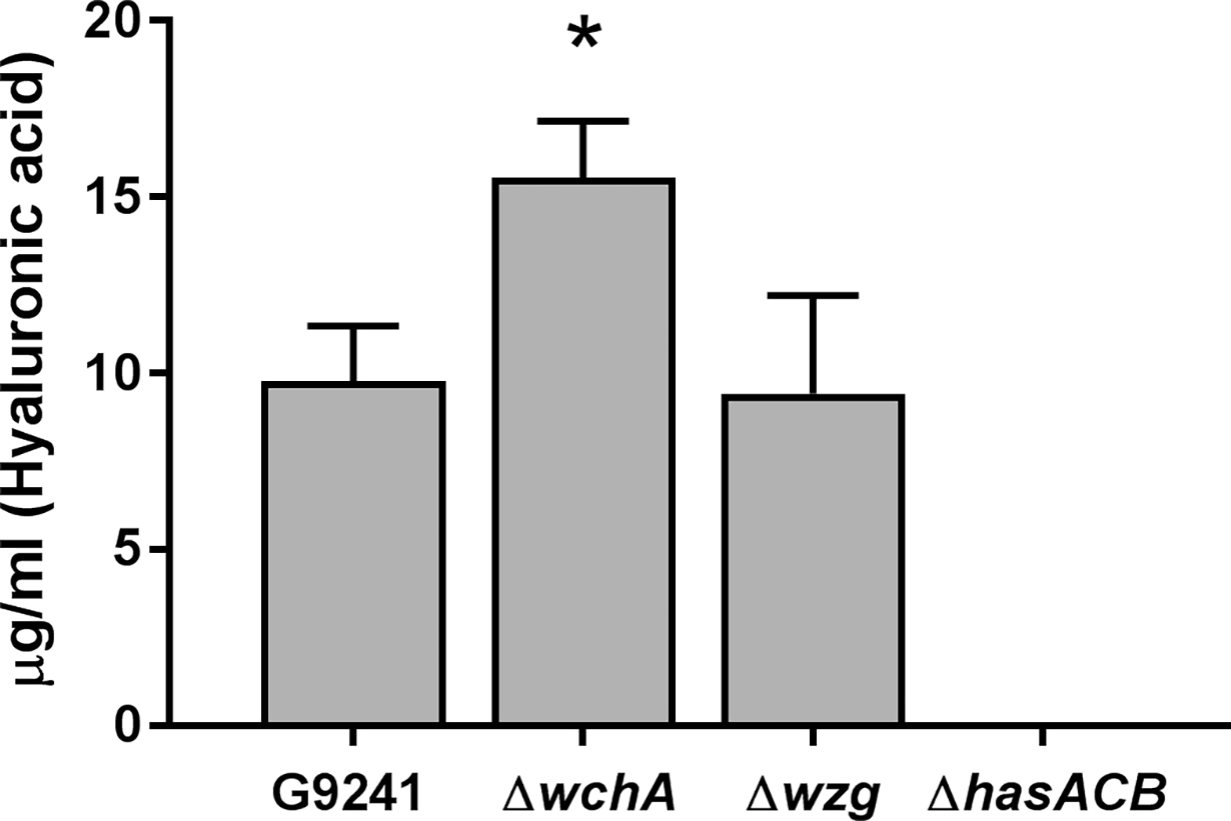
Quantification of HA capsule. The HA capsule was extracted from the cell surface with chloroform from 2 ml G9241, Δ*wchA*, Δ*wzg*, Δ*hasACB*, Δ*wchA*Δ*hasACB*, and pBCXO1^-^/pBC210^-^ cells at OD_600_ 0.34–0.36. The quantity of HA capsule in the extract was determined by Stains-all assay and comparison against a standard curve of purified HA. Three strains (Δ*hasACB*, Δ*wchA*Δ*hasACB* and pBCXO1^-^/pBC210^-^) had no detectable HA by this assay. Only Δ*hasACB* values are shown on the graph. The bars indicate the mean of four experiments and the error bars the standard deviation. * Significantly different (*P* < 0.0003) from G9241 and Δ*wzg* as determined by one-way ANOVA with Tukey’s multiple comparisons test.

To quantify the amount of HA capsule produced by G9241 and the capsule mutants, we extracted the HA capsule from the cell surface with chloroform and quantified the capsule with Stains-all (Fig 6). The Δ*wchA* mutant produced significantly more capsule (15 μg/ml) than either G9241 (10 μg/ml) or Δ*wzg* (10 μg/ml) (*P* < 0.0003). No capsule was detected in the extracts from the strains that did not appear to express HA microscopically: Δ*hasACB*, Δ*wchA*Δ*hasACB*, or pBCXO1^-^/pBC210^-^.

### Effect of capsule mutations on virulence

**Fig 7:**
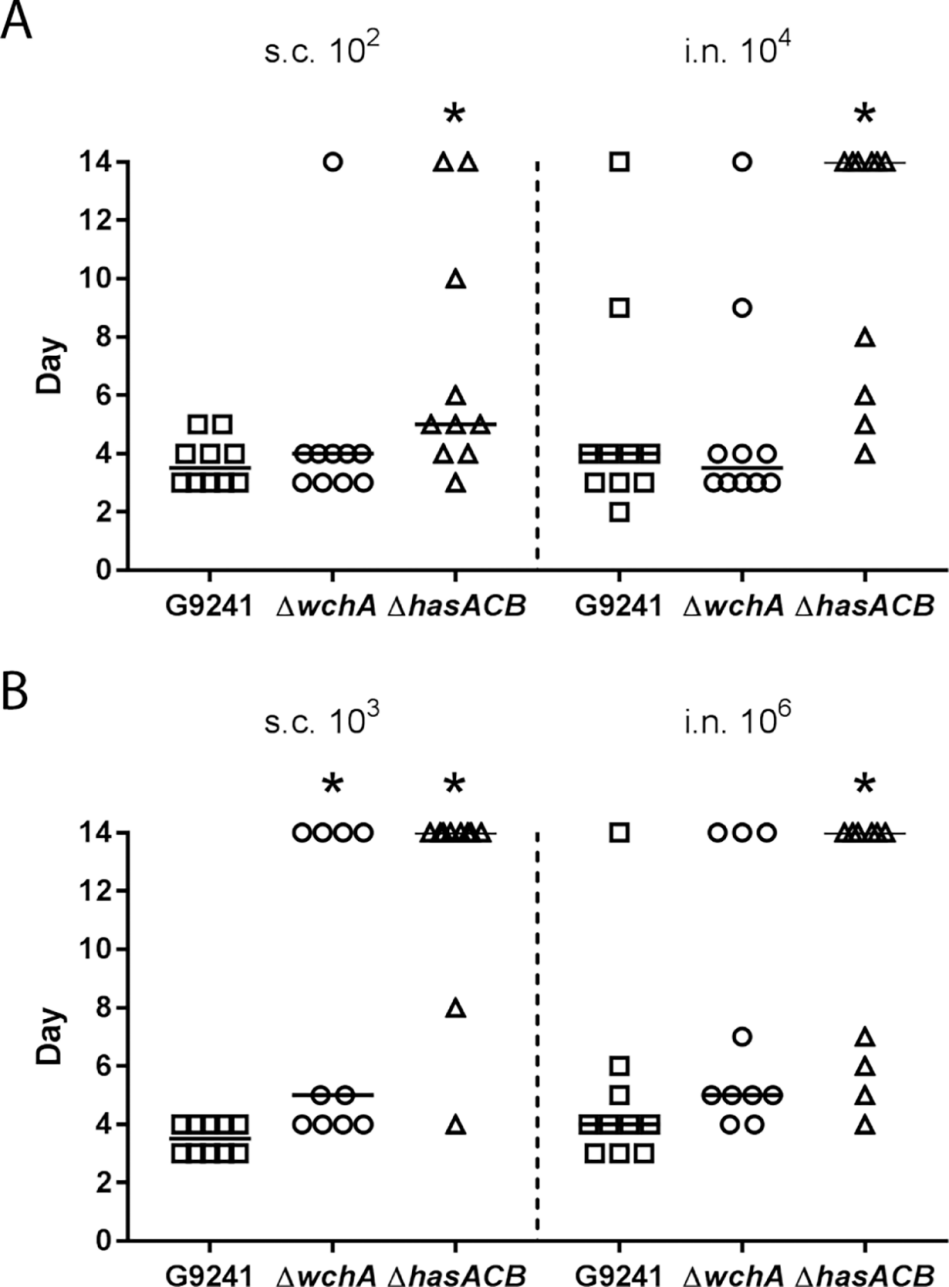
Median survival of A/J and C57BL/6 mice after infection with spores. (A) Day that A/J mice succumbed to infection after inoculation with 10^2^ spores s.c. or 10^4^ spores i.n. of G9241, Δ*wchA*, or Δ*hasACB*. (B) Day that C57BL/6 mice succumbed to infection after inoculation with 10^3^ spores s.c. or 10^6^ spores i.n. of G9241, Δ*wchA*, or Δ*hasACB*. The horizontal black line represents the median, and symbols on day 14 are mice that survived the infection. * Significantly different from G9241 spores at same dose as determined by Kruskal-Wallis with Dunn’s multiple comparisons test.

To determine whether the changes in capsule phenotypes in the mutants also affected virulence, we inoculated mice with spores and calculated the lethal dose 50% (LD_50_) (Table 1). As previously reported, the LD_50_s for G9241 in the more susceptible A/J mice were 7 spores for subcutaneous (s.c.) infection and 3 x 10^3^ spores for intranasal (i.n.) infection [12]. The LD_50_ of Δ*hasACB* was 40 spores for s.c. and 2 x 10^4^ spores for the i.n. infection routes, both of which are significantly different from the LD_50_s of G9241. There was no change in virulence of the Δ*wchA* spores; the LD_50_ values were 8 spores for s.c. infection and 3 x 10^3^ spores for i.n. infection. The Δ*wchA*Δ*hasACB* mutant was attenuated s.c. (LD_50_: 8 x 10^3^ spores) and was avirulent when administered i.n. to A/J mice. We also assessed the mean survival times after an inoculation of about ten times the LD_50_ for G9241: 10^2^ spores s.c. and 10^4^ spores i.n. (Fig 7A). In agreement with the differences in LD_50_ values, we observed a significant delay in time until death and an increase in the number of survivors after infection via both s.c. and i.n. routes with Δ*hasACB* spores (*P* < 0.032), but not with Δ*wchA* spores.

**Table 1:** LD_50_^a^ of spores.

	A/J mice		C57BL/6 mice	
Strain	s.c.	i.n.	s.c.	i.n.
G9241^b^	7	3 x 10^3^	44 (16–100)	4 x 10^5^ (2 x 10^5^–9 x 10^5^)
Δ*hasACB*	44^c^ (11–116)	2 x 10^4^^c^ (7 x 10^3^–5 x 10^4^)	6 x 10^3^^c^ (2 x 10^3^–2 x 10^4^)	3 x 10^6^^c^ (8 x 10^5^–2 x 10^7^)
Δ*wchA*	8 (1–30)	3 x 10^3^ (1 x 10^3^–7 x 10^3^)	5 x 10^2^^c^ (2 x 10^2^–1 x 10^3^)	4 x 10^5^ (1 x 10^5^–1 x 10^6^)
Δ*wchA*Δ*hasACB*	8 x 10^3^^c^ (3 x 10^3^–3 x 10^4^)	>10^7^	>10^7^	N/A

^a^ All LD_50_ values calculated by probit analysis; 95% confidence interval, if provided by SPSS software, in parentheses

^b^ As previously published [12]

^c^ LD_50_ significantly different from G9241 as determined by comparison of relative median potencies in SPSS software

We also tested the mutants in more resistant C57BL/6 mice. As previously reported, the LD_50_s for G9241 spores in C57BL/6 mice were 44 spores for s.c. infection and 4 x 10^5^ spores for i.n. infection. The Δ*hasACB* spores were attenuated compared to G9241, with LD_50_s of 6 x 10^3^ spores for s.c. infection and 3 x 10^6^ spores for i.n. infection. The Δ*wchA* spores were attenuated for s.c. infection (5 x 10^2^ spores) but had similar virulence to G9241 for i.n. infection (4 x 10^5^ spores). The Δ*wchA*Δ*hasACB* spores were only administered to C57BL/6 mice via the s.c. route, and the spores were avirulent. We also compared the median survival of mice after inoculation of ten times the LD_50_ for G9241 in C57BL/6 mice (Fig 7B). There was a significant difference in survival time for mice after s.c. inoculation with 10^3^ G9241 spores compared to mice that received either Δ*hasACB* (*P* < 0.0001) or Δ*wchA* (*P* = 0.0139) spores. After i.n. inoculation, only mice that received 10^6^ Δ*hasACB* spores had a difference in survival time compared to mice that received the same dose of G9241 spores (*P* = 0.0051).

## Discussion

In this study, we characterized the expression of genes in the TS capsule locus and determined that these genes are likely transcribed as two operons. Additionally, we defined the promoter regions upstream of the genes at the beginning of the two operons that were activated under capsule-inducing conditions. We also identified genes in both of these TS capsule operons that are essential for capsule production. Deletion of *wchJ*, a glycosyltransferase, and *wzy*, the polymerase, in the first operon abrogated production of the TS capsule. Similarly, deletion of the initiating glycosyltransferase, *wchA*, in the second operon also abrogated production of the TS capsule. The former two genes were not included in the previous characterization of the TS capsule locus as *bpsX*-*H* [11].

*S*. *pneumoniae* capsule regions have a single promoter upstream of the first gene of the locus. The genes are aligned in close proximity, often with less than 100 bp between genes, and are transcribed as a single operon [28, 29]. Additionally, the locus for *Staphylococcus aureus* type 8 capsule is transcribed as a single operon [30]. However, the G9241 capsule locus consists of two potential operons with a gap of over 1,500 bp between the operons. While the results of the gene junction PCR indicate that *wzg* is the first gene in the potential second transcript, no promoter region was identified upstream of the gene. One possibility is that *wzg* is transcribed from the first operon in the capsule locus and the full length read that includes *wzg* is present at such low levels that we could not detect the junction. The promoter that drives *wzg* transcription could be so weak that the β-galactosidase did not reach levels sufficient to see activity. We did, however, detect promoter activity upstream of *wzd*, the second gene in the potential second transcript. This indicates that if a single transcript of the TS capsule locus is possible, then other promoter sites to initiate transcription along the operon exist.

Both capsule loci contain a gene whose product has the same function (*galU*/*hasC*) which could cause competition for substrates required for the polysaccharide subunits. There was more HA capsule isolated from Δ*wchA* cells, which produce only the HA capsule, than from the G9241 and Δ*wzg* cells that produce both capsules. While we are unable to quantify the TS capsule, the halo visible around Δ*hasACB* cells, which produced only the TS capsule, with Maneval stain was larger than the halos observed post-hyaluronidase treatment of strains that produced both capsules. We speculate that when a single polysaccharide capsule is produced, more substrate is available for the remaining capsule synthesis enzymes to use and more capsule can be produced.

Bacterial capsules help the bacteria evade the immune system by mimicry of host molecules, reduction of phagocytosis, and protection from complement. The disaccharide of the HA capsule, hyaluronan, as well as sialic acid from the TS capsule are both produced by mammalian hosts and might not be immunogenic. Additionally, both the HA and TS capsules inhibit phagocytosis of G9241 by a murine macrophage cell line [11]. The bacterial capsules may also protect the bacteria from complement, a possibility that is consistent with the observation that the non-encapsulated Δ*wchA*Δ*hasACB* strain was avirulent via s.c. infection in C57BL/6 mice but was virulent in A/J mice. A/J mice are naturally deficient in complement component C5 and have reduced inflammatory cell recruitment to the site of infection [31, 32].

In a previous report, C57BL/6 mice had a survival rate of 40% after intraperitoneal challenge with 10^5^ Δ*hasACB* spores and 50% after a dose of 10^5^ spores of a TS-deficient strain [11]. This finding varies from our results that demonstrate a ten-fold difference in virulence between Δ*hasACB* and the TS-deficient Δ*wchA* strain in C57BL/6 mice. The LD_50_ of the Δ*hasACB* spores and TS-deficient spores via intraperitoneal infection were not provided, but it is possible that a 10^5^ dose of spores is much higher than the LD_50_ value, such that differences between the strains would not be apparent. However, even in the more susceptible A/J mice, the Δ*hasACB* mutant had a five-fold increase in LD_50_ after s.c. infection and a ten-fold increase for i.n. infection compared to wild type G9241. These data indicate that the HA capsule is more important for virulence than the TS capsule. In the complement competent C57BL/6 mice, capsule is required for virulence and this is consistent with the previous findings that a non-encapsulated G9241 mutant was avirulent via intraperitoneal infection in C57BL/6 mice [11].

*B*. *cereus* biovar *anthracis* strain CA, isolated from great apes, contains pBCXO1 and pBCXO2 and produces HA and PDGA capsules. When the *hasACB* operon was deleted, there was no change in LD_50_ compared to the wild type strain in outbred mice for either s.c. or i.n. infection routes. When pBCXO2 was cured from the strain and HA was the only capsule produced, there was an almost ten-fold increase in LD_50_ after s.c. infection, but no difference in LD_50_ after i.n. infection [27]. These results suggest that the PDGA capsule is more important for virulence in a s.c. infection than the HA capsule, although it is possible that other factors on pBCXO2 could contribute to virulence. This deficiency in the contribution of the HA capsule to virulence compared to the PDGA capsule could explain the reduced virulence of G9241 compared to *B*. *anthracis* in some models.

Overall, we demonstrated that the TS capsule locus consists of two operons and that genes in both operons are required for G9241 to elaborate the TS capsule. Our data also indicate that if a single capsule is produced, as we demonstrated with the HA capsule, that more capsule may be expressed on the cell surface. With the emergence of *B*. *cereus* strains that produce the *B*. *anthracis*-associated toxins and elaborate capsules that are not the PDGA capsule, it is important to understand how these capsules contribute to virulence. For G9241, we determined that while at least one of its polysaccharide capsules is required for virulence, the presence of the HA capsule may be more beneficial to the bacterial cell in the *in vivo* environment.

## Materials and methods

### Strains and growth conditions

All bacterial strains and plasmids used in this study are listed in Table 2. For growth, bacteria were cultured in Luria-Bertani broth supplemented with antibiotics (when required) as follows: kanamycin (100 μg/ml) or erythromycin (5 μg/ml) for *B*. *cereus* and kanamycin (50 μg/ml), chloramphenicol (20 μg/ml), or ampicillin (100 μg/ml) for *Escherichia coli* unless otherwise specified. Unless otherwise specified, for capsule-inducing conditions, an overnight culture of *B*. *cereus* was sub-cultured 1:1,000 into fresh heart infusion (HI) broth supplemented with 50% heat-inactivated horse serum and 0.8% sodium bicarbonate and incubated at 37°C with 14% CO_2_. Spores were cultivated and isolated as described previously [12].

**Table 2:** Bacterial strains and plasmids.

Name	Description	Reference
*B*. *cereus* strains		
G9241	Wild type strain with pBCXO1 and pBC210	[7]
Δ*wzg*	G9241 Δ*wzg*	This study
Δ*wchA*	G9241 Δ*wchA*	This study
Δ*hasACB*	G9241 Δ*hasACB*::Ω-kan; Kan^r^	This study
Δ*wchA*Δ*hasACB*	G9241 Δ*wchA*Δ*hasACB*::Ω-kan, Kan^r^	This study
Δ*wchJ*	G9241 Δ*wchJ*	This study
Δ*wzy*	G9241 Δ*wzy*	This study
pBCXO1^-^/pBC210^-^	G9241 cured of pBCXO1 and pBC210	[33]
Plasmids		
pUTE304-18Z	Low copy number plasmid pHT304 with promoterless *lacZ*; Ap^r^ in *E*. *coli*, Erm^r^ in *B*. *cereus*	[34]

Ap^r^, ampicillin resistant; Erm^r^, erythromycin resistant; Kan^r^, kanamycin resistant

### RNA extraction, RT-PCR, and 5’ RACE

To determine whether genes for the synthesis of the TS capsule were expressed in an operon, RNA was extracted from cells grown to late-exponential phase (HI broth supplemented with 0.8% sodium bicarbonate, 37°C with 5% CO_2_) with slight modification to a method described previously [35, 36]. Briefly, bacteria were harvested from cultures via centrifugation at 10,000 x *g* for 15 min at 4°C. The pellet was resuspended in 1 ml lysozyme (20 mg/ml in TE buffer) and incubated for 2 h at 37°C, with shaking at 250 rpm. The cells were then lysed by the addition of 200 μl 20% SDS and 10 μl of proteinase K (20 mg/ml). After the samples were incubated for 1 h at 37°C with shaking, 3 ml TRIzol (Thermo Fisher Scientific, Waltham, MA) was added. The samples were agitated with a vortex three times for 5 sec and then allowed to sit at room temperature for 3–5 min. Chloroform (0.2 ml/1 ml TRIzol) was added and the samples were agitated on a vortex for 15 sec. The samples were separated by centrifugation at 3,000 x *g* for 15 min at 4°C, and the top aqueous phase was transferred to a fresh 15 ml conical tube. An equal volume of 100% isopropanol was added, and the samples were shaken with a vortex at low speed. The RNA was subsequently isolated via a vacuum manifold procedure and was eluted with RNase-free H_2_O twice. The RNA was checked for integrity by gel electrophoresis on a 1% agarose and 0.5X TBE gel. The cDNA was then synthesized with the Quantitect Reverse Transcription kit (QIAGEN, Germantown, MD). To test whether the capsule synthesis genes were transcribed together, cDNA was used as the template for PCR-amplification of junctions between genes. A complete list of primers used for this study can be found in S1 Table.

For determination of transcriptional start sites, RNA was extracted with RNAZol as previously described [12] from cultures grown under capsule-inducing conditions to late-exponential phase. DNA contamination was removed from the RNA sample by treatment with the Turbo DNA-*free* kit (ThermoFisher Scientific). To map transcriptional start sites upstream of *wchJ* and *wzd*, RNA was used with the 5’ RACE system for Rapid Amplification of cDNA Ends kit, per the manufacturer’s directions (ThermoFisher Scientific).

### Generation of genetic knockouts

The *hasACB* operon was replaced with an Ω-kanamycin cassette as described previously [37]. Briefly, the 1 kb flanking regions of the operon were cloned, the kanamycin cassette placed between the regions, and the entire fragment was cloned into pUTE583 for electroporation into G9241. Four genes in the pBC210 capsule operon, *wchJ*, *wzy*, *wzg*, and *wchA*, were deleted as described previously [12]. Briefly, the 1 kb flanking regions of the genes of interest were cloned into pJMS1 for electroporation into G9241. A Δ*wchA*Δ*hasACB* double mutant was generated by replacement of *hasACB* with a kanamycin cassette in the Δ*wchA* mutant. Selection of the recombination events was completed as described previously [12, 37]. All mutations were confirmed by PCR and/or Southern blot. The primers used for mutagenesis and confirmation can be found in S1 Table.

### Promoter assays

DNA fragments that contained the upstream region of DNA prior to the ATG start codon for *wchJ*, *wzg*, and *wzd* were cloned into pCR-Blunt II TOPO and confirmed by sequencing the inserted fragments. The primers used to construct the DNA fragments are listed in S1 Table. The fragments were excised with BamHI or BamHI and XbaI, based on the orientation of the fragment in pCR-Blunt II TOPO and ligated into pHT304-18Z in front of the *lacZ* gene [34]. The pHT304-18Z vectors were introduced into Δ*wchA*Δ*hasACB* as described previously [37]. Cells were grown under capsule-inducing conditions in media supplemented with erythromycin (2.5 μg/ml). At 4 h, the OD_600_ was recorded and 2 ml of culture were harvested by centrifugation at 16,000 x *g* for 2 min. Cells were resuspended in 1 ml Z buffer [0.06M Na_2_HPO_4_, 0.04M NaH_2_PO_4_, 0.01M KCl, 0.001M MgSO_4_, 0.05M β-mercaptoethanol] and transferred to 2 ml screw cap tubes with 200–300 μl 0.1 mm zirconia/silica beads. The cells were lysed with the Turbo Mix vortex attachment for 1 min total at setting 9, with 30 s of rest on ice between each 30 s period on the bead beater. Debris was removed by centrifugation at 16,000 x *g* for 10 min. The supernatant was removed and added to a fresh tube in which β-galactosidase activity was assayed as previously described by Miller [38]. Each assay was completed in duplicate four separate times, and significant differences compared to empty vector controls were determined by one-way ANOVA with Dunnett’s multiple comparisons test.

### Hyaluronidase treatment and visualization of capsule

Cells from cultures grown under capsule-inducing conditions for 24 h were fixed in 10% formaldehyde for at least 1 h. The cells were washed 3 times with PBS, and 50 μl of cells were treated with 200 units of hyaluronidase (Sigma-Aldrich) or an equal volume of water at 37°C for 1–2 h. The cells were washed to remove the hyaluronidase and visualized with Maneval stain (Carolina Biological Supply Co., Burlington, NC) as described previously [12]. Briefly, a sample of bacteria was smeared with 1% Congo red and air dried. Maneval stain was applied to the samples for 5 min, then slides were washed with water and air dried. Slides were viewed under oil immersion at 1,000X magnification.

### Chloroform extraction and Stains-all assay

For extraction of HA capsule from cells, cultures were grown for 24 h under capsule-inducing conditions and then 2 ml of cultures standardized to an OD_600_ 0.34–0.36 were harvested by centrifugation for 10 min at 16,000 x *g*. Cells were washed with water and the cell pellet resuspended in 0.5 ml water and transferred to a 2 ml screw cap tube. A 1 ml volume of chloroform was added to the cell suspension, and the reaction was agitated for 5 min on a Turbo Mix vortex attachment at a setting of 2. The aqueous and organic layers were separated by centrifugation at 16,000 x *g* for 10 min. The aqueous (upper) layer was removed and the chloroform extraction repeated. A 25 μl volume of the aqueous layer of the second extraction was mixed with 100 μl of Stains all solution [50% formamide, 6 μl glacial acetic acid and 2 mg Stains-all (Sigma-Aldrich, St. Louis, MO) per 10 ml of solution]. The A_640_ was obtained and compared to values of a standard curve obtained with purified HA from *Streptococcus equi* (Sigma-Aldrich) to determine the concentration (μg/ml) of HA. Significant differences in the production of HA among strains was determined by one-way ANOVA with Tukey’s multiple comparisons test.

### Inoculation of mice with spores

All mouse infections were done under ABSL-2 conditions with approval from the Institutional Animal Care and Use Committee at Uniformed Services University of the Health Sciences (Protocol MIC-15-418) in accordance with the Guide for the Care and Use of Laboratory Animals of the National Institutes of Health. Spores suspended in 100 μl PBS were injected s.c. into the right flank of six-week old female A/J or C57/BL6 mice (Jackson Laboratories, Bar Harbor, ME) or 50 μl of spores in PBS were introduced i.n. to the anesthetized mice as described previously [33]. Groups of five mice were inoculated with spores serially diluted 10-fold. At least two experiments were completed to obtain the data for calculation of the LD_50_ and median survival. Mice were then monitored for morbidity or mortality for two weeks. Humane endpoints were observed when possible. Mice that displayed multiple distress signs, such as ruffled fur, lethargy, hunched posture, and/or lack of response to stimuli, were euthanized by isoflurane overdose followed by cervical dislocation. The LD_50_ was calculated by probit analysis with IBM SPSS Statistics 22 software (IBM, Armonk, NY). Comparisons among the LD_50_s of different strains were completed by comparison of the relative median potency values in SPSS software. If the 95% confidence interval of the relative median potency value did not contain the number one, then the LD_50_s were significantly different. Significant differences in the median survival of mice following infection with spores were determined by Kruskal-Wallis with Dunn’s multiple comparisons test.

## Supporting information

S1 Table Primers used in this study. The sequences of primers and their purpose in this study.
(DOCX)
