# Supplementary material for: Expression and contribution to virulence of each polysaccharide capsule of Bacillus cereus strain G9241
Source: PLoS One. 2018 Aug 22;13(8):e0202701. doi: 10.1371/journal.pone.0202701 (PMC6105005; doi:10.1371/journal.pone.0202701)
Supplement: S1 Table — The sequences of primers and their purpose in this study. (DOCX) [file pone.0202701.s001.docx]

**Table S1. Primers used in this study.**

| Name | Primer Sequence | Use |
| --- | --- | --- |
|  |  |  |
|  | Primers for TS capsule gene junction PCR |  |
| J1 | CCA TTC GTA AAA TTC ATA AAC ATG TCA ATT CAA TCC TC | *wchJ – wzy* junction |
| J2 | GAC TAT TAC TAT ATT CCG GGA ATG GTT GTA AAC CAC |  |
| J3 | CAG CAC TGT TTA AAT GAG ATT TAT ATA TAG AAT CCT TTT GAG C | *wzy* – *wchO* junction |
| J4 | CAA GCA AAT GAT GGA TCA ATA TTC GCA GG |  |
| J5 | GAG CCT CTC TGA CCC CAG TAT G | *wchO* – *wsiK* junction |
| J6 | GCT ATG GGA GTA CCC TTG CAA GAA AC |  |
| J7 | CAG ATG AAG AAG ATT CAT CAC CAA CAA TAA CAA G | *wsiK* – *wzh* junction |
| J8 | GGT TTG AGC TGT GTA GAT TCC TCA GC |  |
| J9 | GTG AGC TGA TGT ATC CAG CTC TTC C | *wsiK* – *wzh* junction |
| J10 | CCA CCG AAT CCA GTC TAC AAT GCG |  |
| J11 | GAT CCA ACG GCT TAT GCA CTG C | *wzh* – *wzg* junction |
| J12 | GGA AGA GCT GGA TAC ATC AGC TCA C |  |
| J13 | GAA TAG CCA TAA TTT CAC GTA GGC TAA CTA TCC | *wzg* – *wzd* junction |
| J14 | CCA AGG TGC TGG CCA TAA AGC |  |
| J15 | GTT CTA ACT ATT CGA TAT TGT TCT GAG ATA GGT GAT TG | *wzd* – *wze* junction |
| J16 | GAT GTG ATG AAT TCA TTA GAA TTA TCT GTT CTG GGT AG |  |
| J17 | CTA GTA CCA AGT CCT GCT GCT GG | *wze* – *galU* junction |
| J18 | CTC AAG TAA TGG CGA ACC AAT GTG ATG G |  |
| J19 | CTT TCG GTC CTA GAA ATC GAA CTA GTA TTT GAC | *galU* – *wchA* junction |
| J20 | GAT TCA ATT AAC GGA TGC GAT TCA ACG ATT G |  |
| J21 | GCC TTA TGG GCA GCA TCC AC | *wchA* – *neuB* junction |
| J22 | CCA GGA TGT ACA GGA CTT TGG C |  |
| J23 | CAA TCT CTC TCC AAG TAG ACC CAT ATT TAG AGA G | *neuB* – *neuC* junction |
| J24 | CAA CAG TTG TGA CCA TAG AAG ATC TAA GGG |  |
| J25 | CAC CTC TTG CAG GAA TTA TGG CTA C | *neuC* – *neuA* junction |
| J26 | GGT GTA CCA TCA GTA GAC ATA GGT AAC AG |  |
| J27 | CTT CAG GAT TAA GGT AAC GAG TTA GTA TAG GAA TC | *neuA* – *wzx* junction |
| J28 | GTG CTG CTA CAA CCA ACA CAA CC |  |
| 5’ RACE Primers | | |
| P1 | GTGGTTTACAACCATTCCCGGAATATAGTAATAGTC | *wchJ* GSP1 |
| P2 | CCAAGTTTCTTCGCCAGATATAAGAATTGTTCC | *wchJ* GSP2 |
| P3 | GCAATATATTCACATTCAGATGCCTTAATAGCCG | *wchJ* nested |
| P4 | CAGGTATTTGTTCACCAGTCTCATCTGC | *wzd* GSP1 |
| P5 | CATTGTCAAGATTCATAATACTAATGACTTCTTCCCG | *wzd* GSP2 |
| P6 | GCAATTCAATTGGATTGTAAACTTCCTGATCAG | *wzd* nested |
|  |  |  |
| Primers for Genetic Knockouts | | |
| P7 | ggatccCCGATTCTCCTCTCATGTTTTGTACCTGG | Δ*hasACB* flanking region 1 |
| P8 | gtcgacGATATACCTAAAGGGTCCAGTATGGGGCG |  |
| P9 | ggatccCAATGATTGAACTGTAACCTATAAAATGGACAGG | Δ*hasACB* flanking region 2 |
| P10 | gcggccgcCGAATTAACAAATTGTGCGGAAAGAACTGC |  |
| P11 | gtcgacGCTACAGTCAATGGATAAGGAAAGGGG | Δ*wchJ* flanking region 1 |
| P12 | ggatccAATCTATTTCTCCTAATTTAAATTTAGTTCAGCAATTCTC |  |
| P13 | ggatccATGTTTATGAATTTTACGAATGGAATTATAGATAAAAATAATACGATTATTTC | Δ*wchJ* flanking region 2 |
| P14 | gagctcCCTGCGAATATTGATCCATCATTTGCTTG |  |
| P15 | ggatccATGAAAGAGAAATTAATAGGAGATATTAAAGTTAATGTAACAAATG | Δ*wzy* flanking region 1 |
| P16 | gagctcGAGCCTCTCTGACCCCAGTATG |  |
| P17 | ggatccGTCAATTCAATCCTCATATCCATATTAATTATTTTTCTTTAACATAC | Δ*wzy* flanking region 2 |
| P18 | gtcgacCAATACAAGCGTTGTTTACTTTGGATAATATCG |  |
| P19 | gaattcCATAACTTATCCCTTCTTTAATATGCACCCATAATTAATTGTAC | Δ*wzg* flanking region 1 |
| P20 | gtcgacCGATTTGTGGTCGGGGTAGAC |  |
| P21 | gaattcGCAGACTGTAATGTAAATGTGAGGAAAAGTGG | Δ*wzg* flanking region 2 |
| P22 | gcggccgcCATTGTCAAGATTCATAATACTAATGACTTCTTCCCG |  |
| P23 | gcggcccgAGTTCACCAGCCCTTAGATCTTCTATGGTCACAACTG | Δ*wchA* flanking region 1 |
| P24 | gaattcTGAAAAAGTGAGCTTCTTGGGAACCAAATTATTCCC |  |
| P25 | gaattcCATTACTTGTACTCCCTAGAAGATTCACTTTTTAATAACCACTCC | Δ*wchA* flanking region 2 |
| P26 | gtcgacGGTTGTTTTAGTTATAATTCCTTGTTTGTATTCTAATAGCATGTTAATG |  |
|  |  |  |
| Primers for Promoter Assay | | |
| P27 | gtcgacGATAGAGGGGAGAGATGGGATGTAGC | *wchJ* 160 bp w/ P12 |
| P28 | gtcgacGGACCGGATAATACTCAAGTTGTTG | *wchJ* 349 bp w/ P12 |
| P29 | gtcgacGAATGCAGAATATCCGTACACGTGATG | *wchJ* 607bp w/ P12 |
| P30 | ggatccAACTTATCCCTTCTTTAATATGCACCC | *wzg* 871bp |
| P31 | gtcgacGTGATATTGATAAAGGTGAGCGGATCAGATTG |  |
| P32 | ggatccAATTTCCTCCTCATCGATCTAACACCTTAATTC | *wzd* promoter |
| P33 | gtcgacCTGTACTTTAGAATTAAGGTGTTAGATCGATGAGG | *wzd* 40 bp w/ P32 |
| P34 | gtcgacCCATTTTATATAGTCTGTGTGGGGATGTTAGC | *wzd* 310 bp w/ P32 |
| P35 | gtcgacGCAGACTGTAATGTAAATGTGAGGAAAAGTGG | *wzd* 502 bp w/ P32 |

Lower case letters are restriction enzyme sites that were added to primer
